# Paternally Expressed *Peg3* Controls Maternally Expressed *Zim1* as a Trans Factor

**DOI:** 10.1371/journal.pone.0108596

**Published:** 2014-09-29

**Authors:** An Ye, Hongzhi He, Joomyeong Kim

**Affiliations:** Department of Biological Sciences, Louisiana State University, Baton Rouge, LA, United States of America; Florida State University, United States of America

## Abstract

The expression of two adjacent imprinted genes, *Peg3* and *Zim1*, is inversely correlated: down-regulation of *Peg3* coinciding with up-regulation of *Zim1*. The current study characterized this inverse correlation using a mutant allele targeting *Peg3*. According to the results, the mutation on the paternal allele of *Peg3* resulted in a dramatic increase in the transcription levels of the maternal allele of *Zim1*, suggesting the involvement of unknown *trans* factors in this trans-allelic event. Subsequent ChIP experiments revealed that the protein encoded by *Peg3* itself binds to the zinc finger exon of *Zim1*, which is modified with the repression mark H3K9me3. Interestingly, the levels of H3K9me3 on *Zim1* are also reduced in the mutant cells lacking the protein PEG3, suggesting potential roles for PEG3 in establishing H3K9me3 on *Zim1*. Reintroducing PEG3 into the mutant cell restored down-regulation of *Zim1*, confirming the predicted repressor role for *Peg3* on *Zim1*. Overall, these results demonstrated that paternally expressed *Peg3* controls maternally expressed *Zim1* as a trans factor. The current study also provides the first case for the trans-allelic interaction of two oppositely imprinted genes through their gene products.

## Introduction

In mammalian imprinted domains, two genes with opposite imprinting are quite often localized right next to each other, and such examples include maternally and paternally expressed *H19*/*Igf2* and *Gtl2*/*Dlk1*. This genomic layout is related to the fact that two genes are usually co-regulated through shared *cis* elements, such as Imprinting Control Regions (ICRs) [1]. As such, one gene is very closely associated with the other gene in terms of their transcription levels and allele-specific expression patterns. This has been well demonstrated through a series of mouse mutagenesis experiments. For instance, mutating an endoderm-specific enhancer located in the 3′-side of *H19* caused down-regulation for both *H19* and *Igf2* [2], yet repositioning this enhancer from the downstream region of *H19* to an intergenic region between the two genes resulted in the reactivation of the silenced maternal allele of *Igf2*, causing biallelic expression of *Igf2* [3]. This has been a basis for identifying an enhancer-blocking function of the ICR that is located immediate upstream of *H19* [4]. It is most likely that similar regulatory mechanisms operate in the other imprinted domains with this genomic layout, the juxtaposition of two adjacent genes with opposite imprinting.

A similar genomic layout is also found in the *Peg3* domain, which contains 7 imprinted genes: paternally expressed *Peg3*, *Usp29*, *APeg3*, *Zfp264* and maternally expressed *Zim1*, *Zim2*, *Zim3* [5]. Among these genes, paternally expressed *Peg3* and maternally expressed *Zim1* are localized right next to each other, suggesting potential co-regulation of these two genes through shared *cis* elements. As expected, this domain is regulated through an ICR, termed the Peg3-DMR (Differentially Methylated Region), the 4-kb genomic region surrounding the 1^st^ exons of *Peg3* and *Usp29* [6]. Deleting part of this ICR, the 2.5-kb genomic region harboring multiple YY1 binding sites, caused global changes in the expression levels and imprinting status of several genes within this domain [7]. In particular, the expression levels of *Peg3* and *Zim1* were affected in a dosage-dependent manner: 4-fold down-regulation of *Peg3* coinciding with 4-fold up-regulation of *Zim1*. Interestingly, the observed up-regulation of *Zim1* was still derived from the maternal allele although the mutation causing down-regulation of *Peg3* was on the paternal allele [7]. This trans-allelic outcome by a mutation has not been observed before, and thus suggests the presence of different regulatory mechanisms involving possible trans factors rather than the known mechanisms involving shared *cis*-regulatory elements as seen from the *H19*/*Igf2* pair.

According to recent studies, *Peg3* encodes a DNA-binding protein with transcriptional repression function [8]. Given the observed tight correlation between *Peg3* and *Zim1*, it is possible that *Peg3* may control directly the transcription of *Zim1* as a trans factor. In this case, the absence or reduced protein levels of PEG3 might be responsible for the observed up-regulation of *Zim1*. To further test this possibility, the *Peg3*/*Zim1* pair was analyzed using a new mutant model targeting *Peg3*. In this new model, the mutation truncates the transcription of *Peg3*, thus removing the PEG3 protein [9]. The results revealed that the removal of PEG3 through the mutation on the paternal allele caused up-regulation of *Zim1* without disrupting its maternal-specific expression. PEG3 also binds to the *Zim1* locus as a trans factor, yet this binding by PEG3 is closely associated with the histone modification mark H3K9me3, suggesting a potential repression mechanism for PEG3. More detailed results have been described in the following sections.

## Results

### Removal of the PEG3 protein results in the up-regulation of *Zim1*

**Figure 1:**
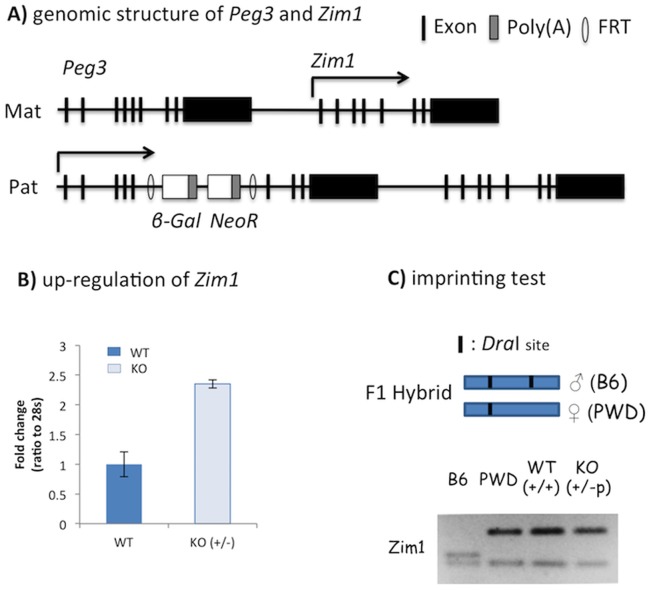
Removal of the PEG3 protein results in the up-regulation of *Zim1*. (**A**) Schematic representation of the genomic structure of paternally expressed *Peg3* and maternally expressed *Zim1*. In the mutant allele, a 7.1-kb cassette containing a promoterless β-galactosidase (*β-Gal*) and human β-actin promoter-driven neomycin resistant gene (*NeoR*) has been inserted between exon 5 and 6 of *Peg3*. The inserted cassette is flanked by two FRT sites (open ovals), thus can be removed through FLP-mediated recombination. (**B**) Expression analyses of *Zim1* using a set of female mouse embryonic fibroblast (MEF) cells that had been prepared through breeding female and male heterozygotes with their wild-type littermates. The MEF cells with the wild-type and the heterozygote with the paternal transmission of the mutant allele were used for qRT-PCR. (**C**) Imprinting tests of *Zim1* using the neonatal brains of the F1 hybrid derived from the crossing of a male heterozygote C57BL/6J (B6) and a female PWD/PhJ (PWD) breeder. The RT-PCR products from *Zim1* were digested with *Dra*I, showing two parental patterns (lane 1 and 2) as well as the maternal-specific expression pattern from the two neonates with WT and KO (lane 3 and 4), respectively. Schematic representation of this imprinting test was shown above the gel picture.

According to the results from the previous study, deletion of part of the Peg3-DMR derived a concurrent 4-fold down and up-regulation of *Peg3* and *Zim1*, respectively [7]. However, it is currently unknown the causal relationship between the observed down and up-regulation of the two genes since the mutation also caused other changes within the *Peg3* domain. To further clarify the observed effects on the *Peg3*/*Zim1* pair, a new mutant model targeting *Peg3* was used for the current study (**Fig. 1A**). This model was originally constructed with a combinatory scheme of knock-in/knock-out, thus will be referred to as a KO model hereafter for the simplicity. In this model, the mutant allele carries an expression cassette containing two ORFs (Open Reading Frames) within its 5^th^ intron of *Peg3*: the promoterless β-galactosidase gene and the neomycin resistance gene driven by the human β-actin promoter [9]. Because of the two poly(A) signals within the cassette, the mutant allele truncates the transcription of *Peg3*, thus removing the PEG3 protein [9]. Subsequent global gene expression analyses revealed that a large number of genes were affected by the removal of PEG3 protein in both the embryos and placentas of 14.5 d.p.c. (days post coitum) [9]. As expected, *Zim1* was also found to be up-regulated in this survey: 2-fold up-regulation in both tissues, which is consistent with the results from the mutant allele deleting part of the Peg3-DMR [7].

To further follow-up this initial result, we have derived a set of mouse embryonic fibroblast (MEF) cells from the mutant animals. Two litters of 14.5-dpc embryos were prepared through timed mating of the female and male heterozygotes for the mutant allele with male and female wild-type littermates, respectively. The first litter inheriting the mutant allele maternally does not have any mutational effects on *Peg3* since *Peg3* is expressed mainly from the paternal allele. In contrast, the second litter inheriting the mutant allele paternally has an effect on *Peg3*, removing the PEG3 protein completely [9]. After genotyping and gender determination, each MEF line from a given embryo was individually cultured, and subsequently used for isolating total RNA for RT-PCR analyses. According to the results from the female set (**Fig. 1B**), the expression levels of *Zim1* were 2.5-fold greater in the paternal heterozyote cell (+/−p) than the wild type cell (+/+). Since this mutant allele disrupts the transcription and translation of *Peg3* only, the observed up-regulation of *Zim1* is most likely an outcome of the removal of PEG3. We repeated this series of expression analyses using another set of MEFs, which also showed a consistent up-regulation of *Zim1* (**Figure S1**). The allele-specific expression of the observed up-regulation of *Zim1* was also tested using the F1 hybrid animals obtained from the inter-specific crossing of C57BL/6J and PWD/PhJ (**Fig. 1C**). The results from the total RNA of the neonatal brain indicated that the up-regulated expression of *Zim1* was still derived from the maternal allele. Overall, this series of expression analyses derived a consistent outcome as seen from the other mutant allele, the up-regulation of *Zim1* on the maternal allele coinciding with the mutation on the paternal allele of *Peg3*. Since both mutant alleles share one common feature, the reduced protein levels of PEG3, the observed up-regulation of *Zim1* is most likely caused by the changed protein levels of PEG3 in both mutant models.

### PEG3 binds to the zinc finger exon of *Zim1*

**Figure 2:**
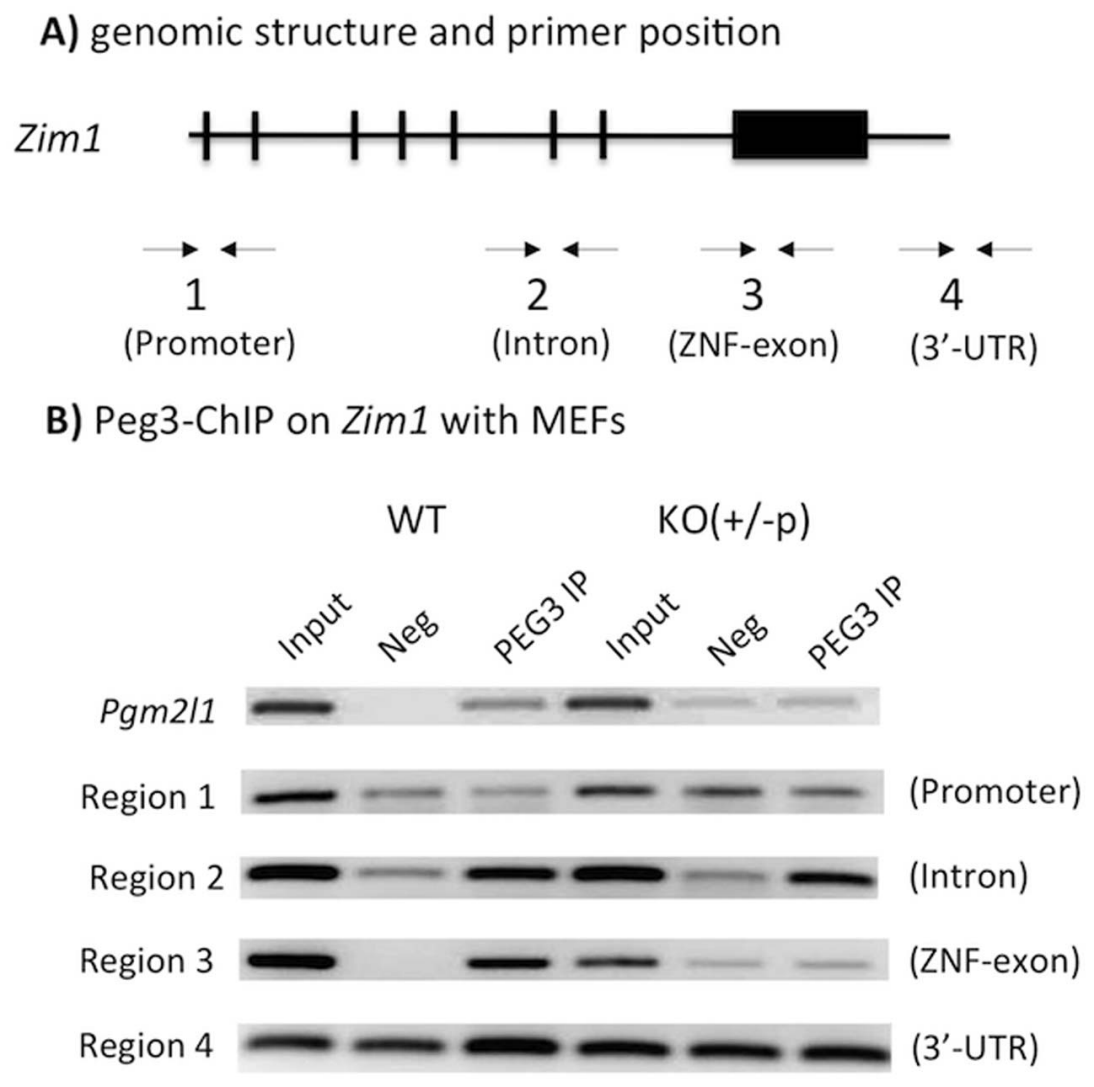
PEG3 binds to the zinc finger exon of *Zim1*. (**A**) Genomic structure of *Zim1* and the relative positions of the primer sets used for ChIP experiments. The 8 exons of *Zim1* are indicated with vertical lines and boxes. (**B**) PEG3-ChIP experiments using the two sets of chromatins prepared from WT and KO (+/−p) MEF cells. The DNA from Inputs, Negative controls (Neg), and Immunoprecipitates with anti-PEG3 antibody (PEG3 IP) was used for PCR amplification. This series of ChIP experiments also included another locus, *Pgm2l1*, as a positive control since this locus is known to be bound by PEG3.

Since *Peg3* encodes a DNA-binding protein with repression activity, we tested the possibility that the protein PEG3 may control directly the transcription of *Zim1* as a trans factor. As an initial step, the binding of PEG3 to the *Zim1* locus was investigated using Chromatin ImmunoPrecipitation (ChIP) technique (**Fig. 2**). For this series of ChIP experiments, 4 genomic regions were selected to scan the entire locus of mouse *Zim1*: Region 1 (promoter), 2 (intron), 3 (zinc finger exon) and 4 (3′-UTR) (**Fig. 2A**). The primer set amplifying the promoter of *Pgm2l1* (phosphoglucomutase 2-like 1) was also included as a positive control since this region has been shown to be a target locus of the PEG3 protein [8]. Three different sets of cross-linked chromatins were immunoprecipitated with anti-PEG3 polyclonal antibodies. First, the immunoprecipitated DNA from the two MEF cells representing wild-type (WT) and mutant (KO) cells were individually analyzed with PCR (**Fig. 2B**). As expected, the enrichment at the *Pgm2l1* locus was detected only in the WT cells but not in the KO cells lacking PEG3, confirming the binding of PEG3 to this locus and also the specificity of our ChIP experimental system. The same set of immunoprecipitated DNA was further tested using the 4 primer sets of *Zim1*. Although the two sets, Region 2 and 4, detected some levels of the enrichment, the detections were not specific to the WT cells, indicating non-specific binding of the anti-PEG3 antibody to other unknown proteins. On the other hand, the promoter region of *Zim1* (Region 1) did not show any level of the enrichment, indicating no binding of PEG3 to this region. In contrast, the zinc finger exon (Region 3) showed much higher levels of the enrichment in the WT cells than in the KO cells, indicating that the observed enrichment likely represents the genuine binding of PEG3 to this region. This has been further confirmed through a set of independent ChIP experiments with a custom-made antibody against PEG3 (**Figure S2**).

**Figure 3:**
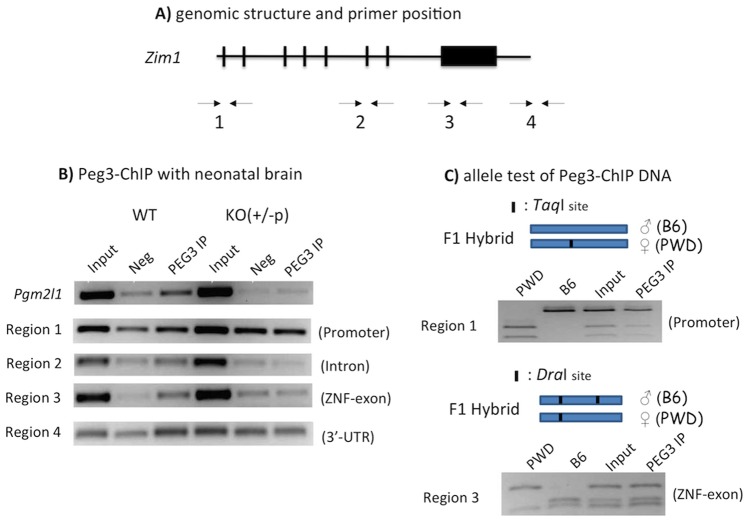
PEG3 binds to both alleles of *Zim1*. (**A**) Genomic structure of *Zim1* and the relative positions of the primer sets used for ChIP experiments. (**B**) Peg3-ChIP experiments using the two sets of chromatins prepared from WT and KO (+/−p) neonatal brains. The DNA from Inputs, Negative controls (Neg), and Immunoprecipitates with anti-PEG3 antibody (PEG3 IP) was used for PCR amplification. This series of ChIP experiments also included another locus, *Pgm2l1*, as a positive control. (**C**) Allele test of PEG3-ChIP DNA. One set of chromatin derived from the F1 neonatal brain of the interspecific crossing between a male C57BL/6J (B6) and female PWD/PhJ (PWD) was used for ChIP experiments. The DNA representing Region 1 and 3 were digested with *Taq*I and *Dra*I, respectively, to differentiate two alleles. The restriction enzyme sites on both DNA fragments are shown along with the schematic representation of these allele tests. The results indicated that the immunoprecipitated DNA at these regions were derived equally from both alleles.

Another set of ChIP experiments was also performed using the second set of chromatin prepared from the brains of the WT and KO (+/−p) neonates (**Fig. 3B**). According to the results, the enrichment at the promoter of *Pgm2l1* was also detected higher levels in the WT than in the KO sample, confirming again the specificity of the anti-PEG3 antibody and the ChIP system. A similar pattern of WT-specific enrichment was observed in both Region 1 and 3, indicating the potential binding of PEG3 to these regions. However, the enrichment at Region 3 is much more obvious than Region 1. Also, the observed enrichment at Region 3 is consistent with the results from the MEF cells, thus confirming the genuine binding of PEG3 to this region in the neonatal brain. Finally, the third set of ChIP experiments were performed using the chromatin prepared from the F1 hybrid between C57BL/6J and PWD/PhJ (**Fig. 3C**). Restriction enzyme digestion scheme was employed to differentiate the two alleles of the immunoprecipitated DNA. According to the results, the immunoprecipitated DNA at Region 1 and 3 both were derived equally from the two parental alleles, indicating that PEG3 likely binds to both alleles of these two regions. Taken together, this series of ChIP experiments concluded that PEG3 binds to the zinc finger exon of *Zim1* on both alleles, further supporting the possibility that PEG3 may control directly the transcription of *Zim1* as a trans factor.

### Reduced levels of H3K9me3 in the mutant cells lacking PEG3

**Figure 4:**
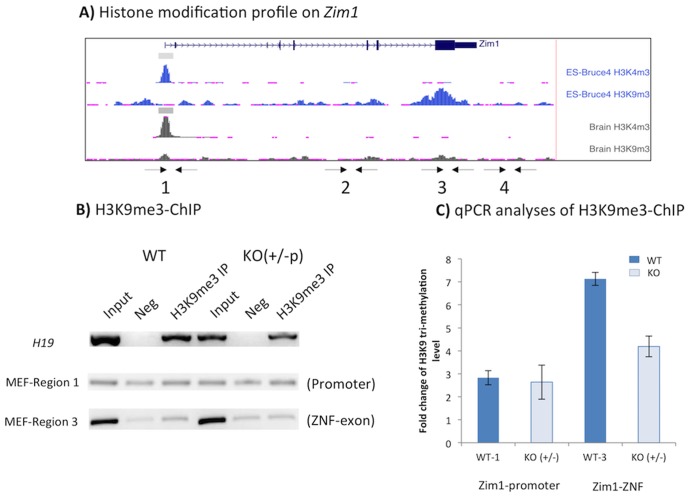
Reduced levels of H3K9me3 in the mutant cells lacking PEG3. (**A**) Histone modification profiles on the *Zim1* locus. The histone modification profiles of H3K4me3 and H3K9me3 derived from ES (upper) and whole brains (lower) are presented along with the exon structure of *Zim1*. (**B**) H3K9me3-ChIP using the two sets of chromatins prepared from WT and KO (+/−p) MEF cells. The DNA from Inputs, Negative controls (Neg), and Immunoprecipitates with anti-H3K9me3 antibody (H3K9me3 IP) was used for PCR amplification. This series of ChIP experiments included another locus, the imprinting control region of *H19*, as a positive control since the paternal allele of this ICR is known to be modified with H3K9me3. (**C**) The reduced levels of H3K9me3 on *Zim1* were further analyzed with qPCR. Shown are the relative values of the immunoprecipitated DNA to the negative controls derived from MEF cells. Region 1 does not show any difference whereas Region 3 shows reduced levels of H3K9me3 in KO compared to those from WT.

The mouse locus of *Zim1* was carefully examined using the genome browser (genome.ucsc.edu) to obtain hints regarding the potential functional outcomes of the observed PEG3 binding. Histone modification levels of H3K4me3 and H3K9me3 derived from ES cells and whole brain tissues are presented as **Fig. 4A**. This examination revealed that the zinc finger exon of *Zim1* is marked with the histone modification H3K9me3 (**Fig. 4A**). Although this histone mark is relatively rare in gene-rich euchromatic regions, it is well known that this modification is quite often associated with the genomic regions encoding the Kruppel-type zinc finger gene family [10]. *Zim1* is a member of this gene family [11], thus the detection of this histone mark at the *Zim1* locus is consistent with the pattern observed from previous studies. This modification at *Zim1* is most obvious in ES cells but some levels of this modification are also detected in other cell types, including adult whole brain. To further test a potential connection between PEG3 binding and H3K9me3, the modification levels of this repression signal were compared between the WT and KO cells. According to the results (**Fig. 4B**), the modification levels of H3K9me3 at Region 3 were 2 fold lower in the KO cells than those in the WT cells although the modification levels at other genomic regions, such as the ICR of *H19*/*Igf2*, were similar between the two MEF cells (**Fig. 4BC**). This indicates that the observed reduction of H3K9me3 in the KO cells is specific to the zinc finger exon of *Zim1*. Since H3K9me3 is known to be a repression mark for transcription, the reduced levels of H3K9me3 is also consistent with the observed up-regulation of *Zim1* in the KO cells lacking PEG3 (**Fig. 1B**). Overall, the genomic region of *Zim1* bound by PEG3 is closely associated with H3K9me3, further suggesting that PEG3 might repress *Zim1* through H3K9me3.

### Restoring the protein levels of PEG3 down-regulates *Zim1*

**Figure 5:**
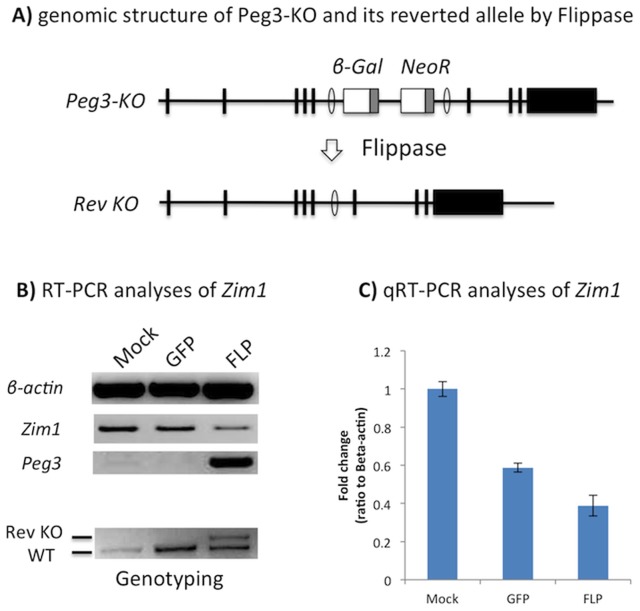
Restoring the protein levels of PEG3 down-regulates *Zim1*. (**A**) Genomic structure of the mutant allele of *Peg3* and FLP-mediated recombination scheme to restore the expression of *Peg3*. The inserted cassette is flanked by two FRT sites, thus can be removed by Flippase (FLP). (**B**) Three pools of KO MEF cells were transfected with the following constructs: no vector as a mock control (lane 1), a Green Fluorescent Protein (GFP) expression vector as a negative control (lane 2), and a FLP expression vector (lane 3). The total RNA isolated from these cells were analyzed with RT-PCR to measure the expression levels of *β-actin*, *Zim1*, and *Peg3*. The bottom panel shows genotyping results confirming FLP-mediated recombination of the mutant allele (Rev KO) and endogenous allele (WT) of *Peg3*. (**C**) The observed down-regulation of *Zim1* was further analyzed using qRT-PCR.

To further test PEG3's repressor role in the transcription of *Zim1*, we performed the following set of *in vitro* transfection experiments (**Fig. 5**). In the MEF KO (+/−p) cells, the transcription of *Peg3* is disrupted by the inserted cassette, which is flanked by two FRT (Flippase Recombination Target) sites. The vector construct expressing Flippase (FLP) was transiently transfected into the KO cells to remove the cassette, restoring the transcription and translation of *Peg3*. This pool of cells was used for measuring the expression levels of *Zim1* along with a set of control cells: the cells transfected with no vector (Mock) and a Green Florescent Protein vector (GFP) (**Fig. 5**). As shown in **Fig. 5B**, the transient expression of FLP indeed removed the inserted cassette based on the detection of a genomic fragment without the inserted cassette. This removal of the cassette subsequently restored the expression of *Peg3* based on RT-PCR. In these cells with the restored PEG3, the transcriptional levels of *Zim1* was 2.5 and 1.5-fold reduced as compared to those observed from the two control cells (**Fig. 5C**). It is also prudent to mention that the transcriptional levels of *Zim1* were further reduced in the set transfected with FLP than in the set with GFP. These results demonstrated that the restored expression of *Peg3* is responsible for the down-regulation of *Zim1*, confirming again the inverse correlation between *Peg3* and *Zim1*. Down-regulation of *Zim1* was further tested with the over-expression of *Peg3* (**Figure S3A**). The results confirmed the down-regulation of *Zim1* (**Figure S3B**). Overall, this series of transfection experiments confirmed that PEG3 functions as a repressor for the transcription of *Zim1*.

## Discussion

In the current study, we tested the possibility that paternally expressed *Peg3* may control the transcription of maternally expressed *Zim1* as a trans factor. According to the results, the reduced protein levels of PEG3 is indeed responsible for the up-regulation of *Zim1*. The PEG3 protein also binds to the zinc finger exon of *Zim1* that is marked with the repression mark H3K9me3. Furthermore, the KO cells lacking PEG3 have the reduced levels of H3K9me3 at the zinc finger exon of *Zim1*, suggesting that PEG3 might control *Zim1* through H3K9me3. *In vitro* transfection experiments further demonstrated that reintroducing the PEG3 protein into the KO cells restores the down-regulation of *Zim1*. Overall, these results confirm that paternally expressed *Peg3* controls maternally expressed *Zim1* as a trans factor.

**Figure 6:**
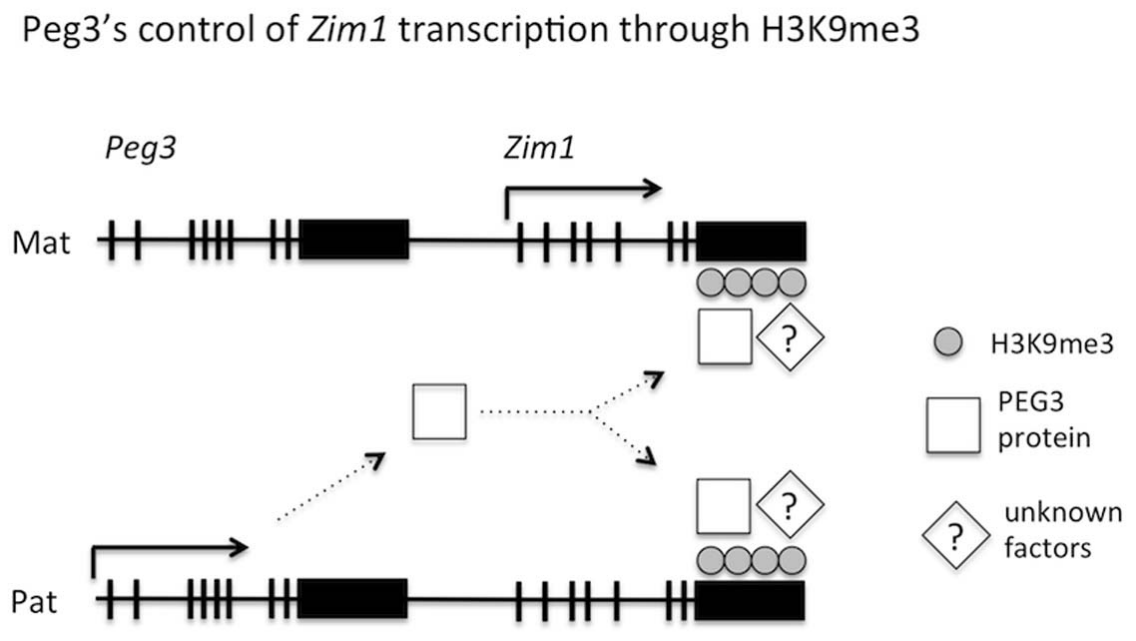
Paternally expressed *Peg3* controls maternally expressed *Zim1* as a trans factor involving H3K9me3. Schematic representation for Peg3's functional role in transcriptional control of *Zim1*. The gene product of paternally expressed *Peg3* binds to the zinc finger exon of maternally expressed *Zim1* on both alleles, resulting in transcriptional repression through H3K9me3. The protein PEG3 may interact with some unknown proteins, such as KAP-1, to recruit SETDB1 for the H3K9me3 modification on the *Zim1* locus. This role of *Peg3* is independent of the maternal-specific expression of *Zim1*, thus the observed up-regulation of *Zim1* is still derived from the maternal allele of the *Peg3* mutant animals.

The imprinted gene pair of *Peg3*/*Zim1* is unique based on the following reasons. As seen in the other pairs of oppositely imprinted genes, the transcriptional level of *Peg3* is also inversely correlated with those of *Zim1* (**Fig. 1B** and **Fig. 5**). However, this inverse correlation does not involve any change in their allele-specific expression pattern (**Fig. 1C**), which is quite different from the other pairs of oppositely imprinted genes [1]. This suggests the involvement of some unknown trans factors in the inverse correlation between *Peg3* and *Zim1*. The results form the current study further indicated that the protein encoded by *Peg3* itself is likely this unknown trans factor (**Fig. 2** and **Fig. 3**). According to previous studies, *Peg3* encodes a DNA-binding protein with repression activity [8]. Also, there is a very tight inverse correlation between *Peg3* and *Zim1*: 4-fold down regulation of *Peg3* coinciding with 4-fold up-regulation of *Zim1* [7]. This line of evidence supports the idea that *Peg3* controls the transcription of *Zim1* as a trans factor. Nevertheless, the results from the current study also confirm another important aspect of *Zim1* that PEG3 is unlikely involved in regulating the allele-specific expression of *Zim1* since the removal of PEG3 does not have any effect on the maternal expression of *Zim1*. The observed PEG3-mediated regulation of the transcriptional levels of *Zim1* appears to be separate from some unknown mechanisms controlling the maternal-specific expression of *Zim1*. In summary, the transcription of *Zim1* is regulated through two separate mechanisms: one controlling the allele-specific expression and the other controlling the expression level through PEG3 (**Fig. 6**).

The binding of PEG3 to the zinc finger exon of *Zim1* is consistent with several known facts about the evolutionary origin of *Peg3* as well as the repression mark H3K9me3. First, *Peg3* is localized in the middle of a Cys2His2-type zinc finger gene cluster [12], yet *Peg3* itself encodes a DNA-binding protein with C2H2 zinc finger motifs [8]. This suggests that *Peg3* may have originated from this type of zinc finger genes (ZNFs). The C2H2-type ZNFs are known to interact the H3K9 methylase SETDB1 *via* the co-repressor protein KAP-1 [13]. Thus, the binding of PEG3 to the genomic region with the H3K9me3 modification makes sense given the evolutionary origin of *Peg3*, and further implies that PEG3 might still recruit SETDB1, possibly through the interaction with KAP-1 (**Fig. 6**). Second, it is well known that ZNFs, such as *Zim1*, are usually regulated through H3K9me3 [10]. This is further supported by the fact that the zinc finger-coding region of *Zim1* is indeed modified by this repression mark in ES and other somatic cells (**Fig. 4A**). Yet, the KO cells lacking PEG3 have the reduced levels of H3K9me3 (**Fig. 4B**). This further suggests that PEG3 likely controls the transcription of *Zim1* through H3K9me3. According to the results from the mutant mouse model targeting *Peg3*, many placenta-specific gene families are also up-regulated in the brains of KO mice, yet all of these gene families are known to be regulated through similar repression mechanisms involving H3K9me3 [9]. Therefore, it is most likely that PEG3's regulation on *Zim1* may be also through H3K9me3.

## Materials and Methods

### Ethics Statement

All the experiments related to mice were performed in accordance with National Institutes of Health guidelines for care and use of animals, and also approved by the Louisiana State University Institutional Animal Care and Use Committee (IACUC), protocol #13-061.

### Derivation of MEF (Mouse Embryonic Fibroblast) cells

Two litters of 14.5-dpc embryos were harvested through timed mating of the male and female mutant mice heterozygous for the KO allele with the female and male wild-type littermates, respectively. The mutant allele of *Peg3* used for the current study has been previously reported and characterized in detail [9]. The head portion and the red tissues were removed from the embryos, and the remaining portions were minced with razor blades. These minced tissues were transferred to a 15 mL conical tubes containing 1 mL trypsin (Invitrogen, Cat. No. 25300062). After 5 min incubation at 37°C, the cells were harvested with centrifugation, and later resuspended in 15 mL media (Life technologies, Cat. No. 10566024). Finally, the resuspended cells were plated onto a T-75 flask. MEF from each embryo was first genotyped using the following primer set: Peg3-for (5′-ATGAGTCTCGATCCCAGGTATGCC-3′) and LoxR (5′-TGAACTGATGGCGAGCTCAGACC-3′). The gender of each MEF was also determined using the following primer set: mSry-F (5′-GTCCCGTGGTGAGAGGCACAAG-3′) and mSry-R (5′-GCAGCTCTACTCCAGTCTTGCC-3′).

### Chromatin ImmunoPrecipitation (ChIP) analyses

Chromatins were prepared from two different types of samples, MEF and neonatal brains, according to the method previously described [8]. In brief, the homogenized samples were first cross-linked with 1% formaldehyde for 20 mins, and then lysed with the buffer containing protease inhibitor cocktail (Millipore, Cat. No. 539131). The released nuclei were fractionated with sonication to a pool of DNA fragments size-ranging from 300 to 1,000 bp in length. The prepared chromatin was immunoprecipitated with the following two antibodies: PEG3 (Abcam, Cat. No. ab99252) and H3K9me3 (Abcam, Cat. No. ab8898). Each immunoprecipitated DNA was dissolved in 100 µl of TE for either PCR or qPCR analyses.

### Transfection experiments

MEF cells were transfected with the following two constructs, GFP (pIRES-puro-GFP) and FLP (pIRES-puro-FLP), using the GenJect transfection reagent (Cat. No. SL100489-MEF) according to the manufacturer's protocol. Transfection efficiency was monitored through GFP expression under a fluorescence microscope after 24 hours. The transfected cells were harvested at 72-hour post transfection for RNA and DNA isolation. The reverted allele of *Peg3* by FLP was detected through PCR with the following primer set: Flpko-F (5′-CCCTCAGCAGAGCTGTTTCCTGCC-3′) and Flpko-R (5′-AAGCTACCTGGGAAATGAGTGTGG-3′).

### RNA isolation and qRT-PCR analyses

Total RNA was isolated from either MEF or the brains of one-day-old neonates using a commercial kit (Trizol, Invitrogen) according to the manufacturer's protocol. The total RNA was then reverse-transcribed using the M-MLV kit (Invitrogen), and the subsequent cDNA was used as a template for quantitative PCR. The qRT-PCR analysis was performed with SYBR Select Master Mix (Applied Biosystems, Life Technologies) using the iCycler iQTM multicolor real-time detection system (Bio-Rad). All qRT-PCR reactions were carried out for 40 cycles under standard PCR conditions with internal controls (*28S* and *β-actin*). The results derived from qRT-PCR were further analyzed using the threshold (Ct) value. The ΔCt value was initially calculated by subtracting Ct value of a testing replicate of a given gene from the average Ct value of the internal control (*28S* and *β-actin*). The fold difference for each replicate was then calculated by raising the ΔΔCt value as a power of 2 [14]. The average and standard deviation for each sample were then calculated by compiling the normalized values. The information regarding individual primer sequences is also available (**Data S1**).

## Supporting Information

Figure S1 **(A) A series of RT-PCR analyses using another set of MEF cells show a consistent up-regulation of** ***Zim1*** **by the mutation on** ***Peg3*** **.** This analysis was performed using two internal controls, 28S and β-actin. (**B**) The up-regulation of *Zim1* in KO MEF cell was further analyzed using qPCR.
(JPG)

Figure S2 (**A**) **A set of independent ChIP experiments with a custom-made anti-PEG3 antibody using the two sets of chromatins prepared from WT and KO (+/−) MEF cells.** The DNA from Inputs, Negative controls (Neg), and Immunoprecipitates with anti-PEG3 antibody (PEG3 IP) was used for PCR amplification. This series of ChIP experiments also included another locus, *Pgm2l1*, as a positive control. (**B**) qPCR analyses using these ChIP DNA derived from MEF cells. Regions 2 and 3 showed some levels of the enrichment, but Region 3 showed the highest enrichment levels. However, no significant enrichment was detected in Region 1. (**C**) Western blotting testing the specificity of a new custom-made antibody using the two sets of total protein prepared from WT and KO (+/−) MEF cells.
(JPG)

Figure S3 **(A) Three pools of KO MEF cells were transfected with the following constructs: No vector as a mock control (lane1), PEG3 expression vector (Lane2), and Green Fluorescent Protein (GFP) expression vector as a negative control.** The total RNA isolated from these cells were analyzed with RT-PCR to measure the expression levels of *β-actin*, *Zim1* and *Peg3*. (**B**) The observed down-regulation of *Zim1* was further analyzed using qRT-PCR.
(JPG)

Data S1 **List of the primers that were used for ChIP and qRT-PCR experiments.**
(DOCX)
