# Supplementary figures and images for: Paternally Expressed Peg3 Controls Maternally Expressed Zim1 as a Trans Factor
Source: PLoS One. 2014 Sep 29;9(9):e108596. doi: 10.1371/journal.pone.0108596 (PMC4180786; doi:10.1371/journal.pone.0108596)

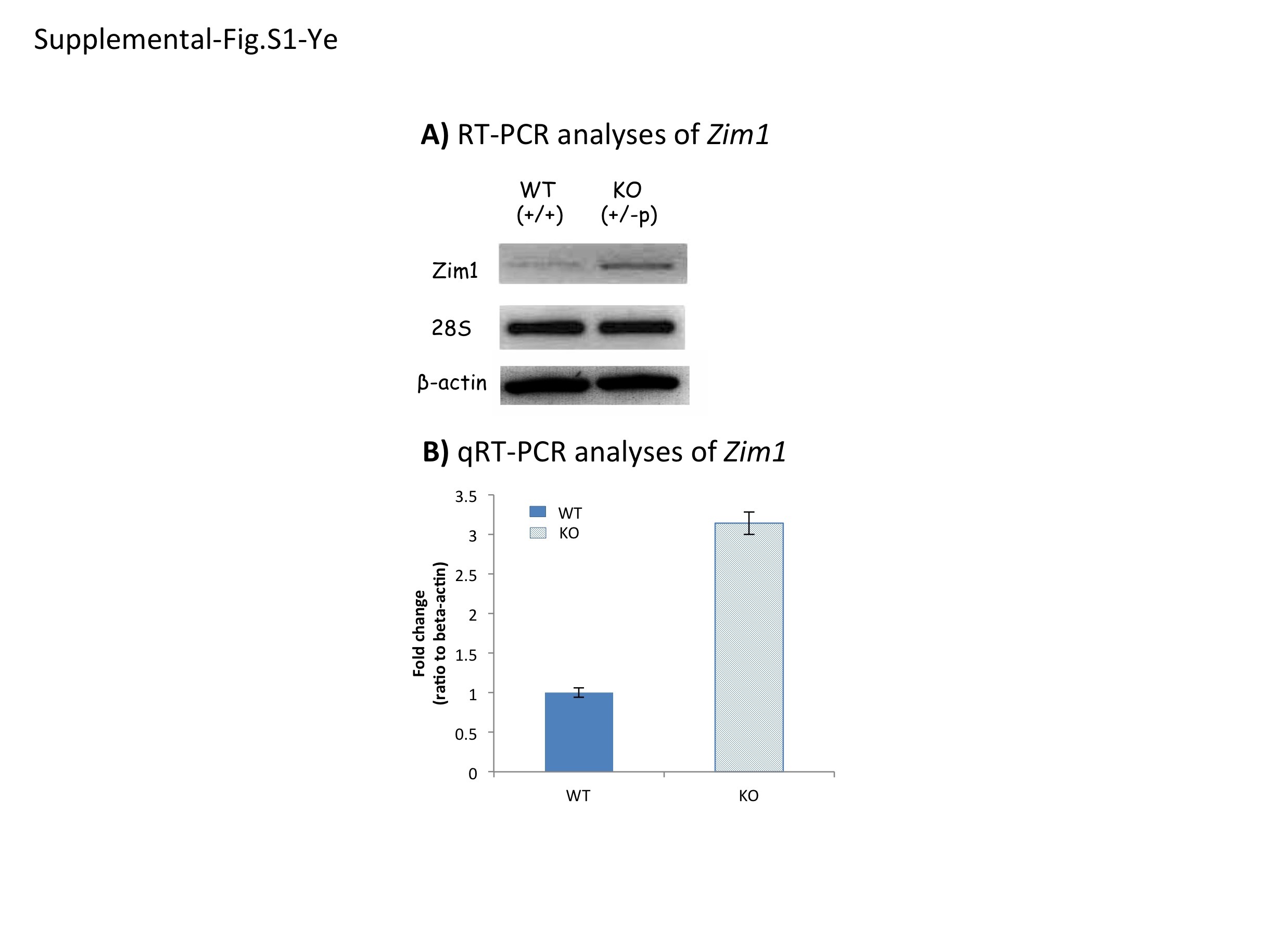

Supplement: Figure S1 — (A) A series of RT-PCR analyses using another set of MEF cells show a consistent up-regulation of Zim1 by the mutation on Peg3 . This analysis was performed using two internal controls, 28S and β-actin. (B) The up-regulation of Zim1 in KO MEF cell was further analyzed using qPCR. (JPG) [file pone.0108596.s001.jpg]

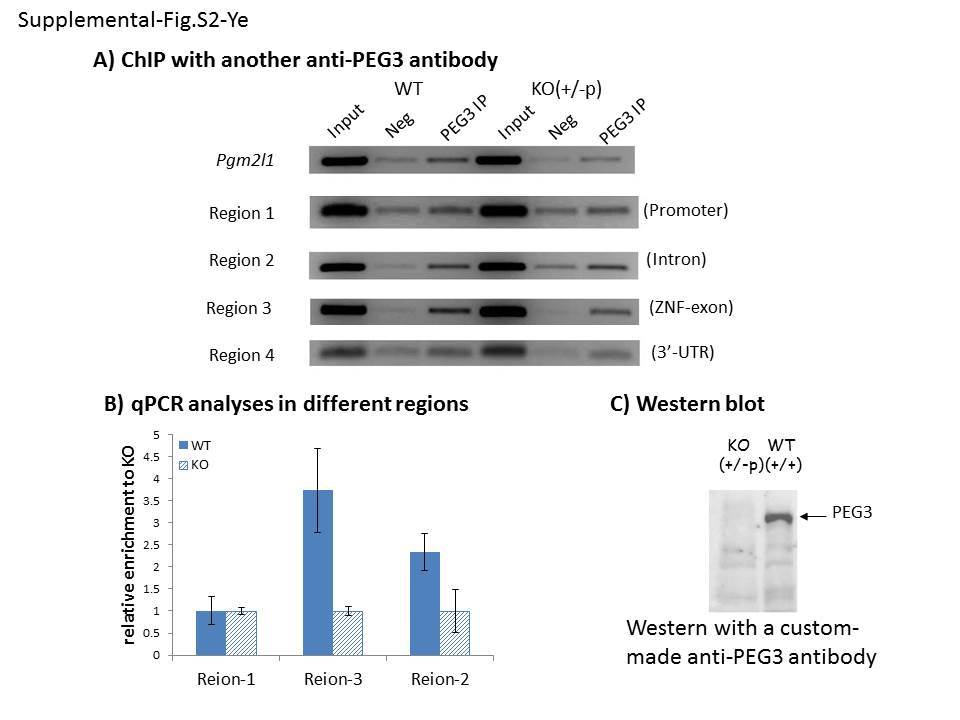

Supplement: Figure S2 — (A) A set of independent ChIP experiments with a custom-made anti-PEG3 antibody using the two sets of chromatins prepared from WT and KO (+/−) MEF cells. The DNA from Inputs, Negative controls (Neg), and Immunoprecipitates with anti-PEG3 antibody (PEG3 IP) was used for PCR amplification. This series of ChIP experiments also included another locus, Pgm2l1, as a positive control. (B) qPCR analyses using these ChIP DNA derived from MEF cells. Regions 2 and 3 showed some levels of the enrichment, but Region 3 showed the highest enrichment levels. However, no significant enrichment was detected in Region 1. (C) Western blotting testing the specificity of a new custom-made antibody using the two sets of total protein prepared from WT and KO (+/−) MEF cells. (JPG) [file pone.0108596.s002.jpg]

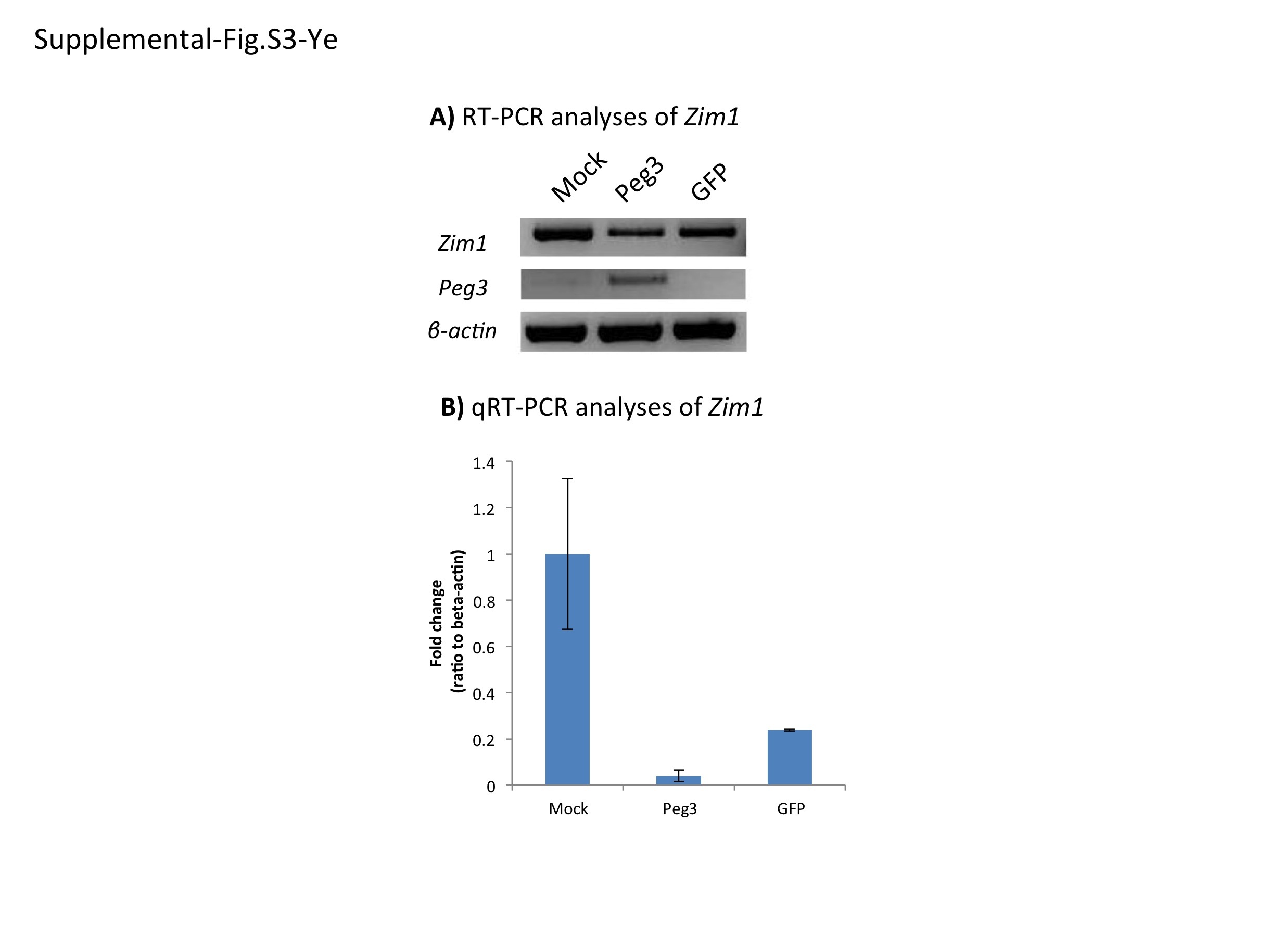

Supplement: Figure S3 — (A) Three pools of KO MEF cells were transfected with the following constructs: No vector as a mock control (lane1), PEG3 expression vector (Lane2), and Green Fluorescent Protein (GFP) expression vector as a negative control. The total RNA isolated from these cells were analyzed with RT-PCR to measure the expression levels of β-actin, Zim1 and Peg3. (B) The observed down-regulation of Zim1 was further analyzed using qRT-PCR. (JPG) [file pone.0108596.s003.jpg]
