# Supplementary material for: Paternally Expressed Peg3 Controls Maternally Expressed Zim1 as a Trans Factor
Source: PLoS One. 2014 Sep 29;9(9):e108596. doi: 10.1371/journal.pone.0108596 (PMC4180786; doi:10.1371/journal.pone.0108596)
Supplement: Data S1 — List of the primers that were used for ChIP and qRT-PCR experiments. (DOCX) [file pone.0108596.s004.docx]

**ChIP Primers;**

Zim1 primer sets

Region 1

Fp------CTCGTCCTCGCGCTTGGTTAT

RP------CCTAGAGCTGGGGACTTGGGTTT

118bp

CTCGTCCTCGCGCTTGGTTATCCGTCCGTCCGCACTCGCCTCCAGGCACCGCAGCCTGCGGCC

ACCCGGAGTGGAATGCCGCGGGATGACTTTCAAAACCCAAGTCCCCAGCTCTAGG

Region 2

FP----TCCCGAGACACATTCCAGCTTCT

RP----GACGCTGTTGACGGCATCCTAA

100bp

TCCCGAGACACATTCCAGCTTCTACTCTGCATACGAGTTAAACTCTGAGCTTCTGAAGAGCA

GGGCTCTTGGAAAGTCTTAGGATGCCGTCAACAGCGTC

Region 3

FP------CATCACAGGCTCCAGCACATTCA

RP----CCCTTCATGGGGTGACTCCAAG

137bp

CATCACAGGCTCCAGCACATTCACAAACCTTACAAAATGCTACATTATGCTGTTAAAAAAAT

TTTTTTAAAAAAGCACAGATAGATTCAGTTTCACAAAGCTGTGTCCTATTTTCCTTGGAGTCA

CCCCATGAAGGG

Region 4

FP-----GGGTGTGGTGGTACATGCCTTTAA

RP-----GACCAGGCTGTCCTTGAACTCTCA

83bp

GGGTGTGGTGGTACATGCCTTTAATCCCAGTACTTAAGAGGCAGAGATGGGAGGATCTCTGA

GAGTTCAAGGACAGCCTGGTC

**Genomic primers**

Zim1 F1/F2

FP----GGATTCACACGGGAGTGAGA

RP----CTTGCACCGGTACCTGGAGT

539bp

GAATTCACACGGGAGTGAGACCCTACAAGTGTAAGGAGTGTGGCAAAGCCTTCAACAGCAG

CTCCACTCTCAACAATCACTGCAGGATCCACTCAGGGGAGAAACCCTTTAAATGTGACGAGT

GCGGGAAAACCTTCAAGCAGAGCACAAAGCTCACCCGTCACCAGCGGATTCATACCGGGGA

GAAGCCCTACAAGTGTGGAGAGTGTAACAAGTGCTTCGGCCGCAGCTCATCCCTGAGGGAG

CACAAGAGGATCCATACCGGAGAGAAGCCGTACTGCTGTCAGGTGTGTGGGAAGACCTTCA

GGGTCAACTCACATCTTTCGGAGCATCAGCGGCTCCATTTAAAGGTGAAGCCGTACAAGTGT

GACAAGTGCGGAAAACACTTCCGAAACAGCTCGTACTTGACGGAGCACAAGCAGATCCATG

TGCCTGGGGCCCGAGTGGACTGCCCGGAGTGTGGGAAAGTCTTCGCTTGCAAAGTAGCCCTT

CTCAAGCACCAGAAGAGACACGAGGCAAACTCCAGGTACCGGTGCAAG

Zim1 a/b

FP----CCCAGACCACTGTCCTCTACAGAG

RP----CCTCAGCGTGTCATCACATAATC

517bp

CCCAGACCACTGTCCTCTACAGAGCTGTGATAGGGTGGGGACCCACCCAGCATGCAGACAC

CCTGCCCTCTCAGTCCTCTCCCGTGTTCTTGCCTCTCGTCCTCGCGCTTGGTTATCCGTCCGTC

CGCACTCGCCTCCAGGCACCGCAGCCTGCGGCCACCCGGAGTGGAATGCCGCGGGATGACT

TTCAAAACCCAAGTCCCCAGCTCTAGGGCACCGCCCACAGGACTACATGTCCCAGAAGGTCT

CACGCCGTCCAGCCTGAGGCTCCAGGCTATGGAGCGCCCAGCACCCGGCACAGCGCGCTGG

GACCTGTGGGAAATGTAGTCCCCAGCAAGATGCGCTCGCTATAAGCGTTCCTCAGACGCTCT

GCCCTTCTGTGGCTCCACTTCTAGTACTATAGAGAAATTGCGTCTCCTGGTGGAAAAATTTCC

TTCGGTAGTAAAATTTCTAAATTGATAGAGGCCGATGGTAGAAAAGGGAAAGATTTTTAAG

ATTATGTGATGACACGCTGAGG

PGM2l1

FP----ACCAAGCCCTGAGGCGAAGA

RP----TGCCGTTCCTCAGCTGTCTTTC

155bp

ACCAAGCCCTGAGGCGAAGAGGGTGGGGCCTGCGCGCGCCGGGGACTCTCCTAGTGAGTGC

AGAGGCGGGCTGCTTGAGACGACGGGGACACCAGGTGGAGGCGCACGTGCGAGTGCGGGG

CGGGTGATAAGGGAAAGACAGCTGAGGAACGGCA

H19

FP---- GTGTGGCTCACTATAGGAAGGCA

RP----CCTATGCCGCGTCTGCCGAGCAA

565bp

GTGTGGCTCACTATAGGAAGGCATAGAAGCTGTTATGTGCAACAAGGGAACGGATGCTACC

GCGCGGTGGCAGCATACTCCTATATATCGTGGCCCAAATGCTGCCAACTTGGGGGGAGCGAT

TCATTCCCAGCAATATCCCAGGGTCACCCAAATAGGGATTCATAGGGGTGGTAAGATGTGTG

CACCTCTGGAATGGTTCCCTTACACACTGAACCAGAGAACTTGACTCATTCCCTACACAGCC

CGAGATCGTCAGTGGCTGGTAAGACCGAAGTTGCCGAGCAGCGACCAGTGCAGTCCCACAT

ACTTTATCATAGAGGTGACCAAAATTGCGGTTCACCTATGGCAAACTCATGGGTCACTCAGG

CATAGCATTCAATGATTCATAAGGGTCATGGGGTGGTACAACACACATTTCTTGGGTAGCTC

CTTCAGTCTTGCGCCCTTCACGATCGATCGGTTCACTCTCCACGCTGTGCAGATTTGGCTATA

GCTAAATGGACAGACGATGCCGCGTGGTGGCAGTACAATACTACATATTGCTCGGCAGACG

CGGCATAGG

FlpKO

FP---- CCCTCAGCAGAGCTGTTTCCTGCC

RP---- AAGCTACCTGGGAAATGAGTGTGG

**RT-Primers;**

Zim1

FP----GATCACCAGGTTGGAGCAAGGAGT

RP----AGCGCTCTGTGGTGTTGTAGTTG

403bp

GATCACCAGGTTGGAGCAAGGAGTTGACCTTTTTGCCAAAGAGAACGATGTTCCTGGAGACC

CCCAGCAAGGTGAGGCAGGAGTCAGCAGAAGTGACACCTCTGCAGGGAAGAAGACAGGGG

ATAATTCCACAAAGGCAGAAATCCGGAAGCCAGACAATTCTAAGATAACATCACTGGAGAA

GCAGAAGGCGGCAGATCAAGGCCGTGGTTCCCAGAGTTTGAGAGCAGAAAAGACCTCAAAA

AGTGATGACAGGCCATCTCAAAATAAGGAGAAATGTGCCTCAACTTCGACTACAGAAGCAA

GCAAAACCAGCATACCGGGCAATAAGGAGAATGAATCTGCAATTCCAGGCACATCTTCGGG

TCAAACATCAGCAGCAACTACAACACCACAGAGCGCT

28s

FP----TTGAAAATCCGGGGGAGAG

RP----ACATTGTTCCAACATGCCAG

100bp

TTGAAAATCCGGGGGAGAGGGTGTAAATCTCGCGCCGGGCCGTACCCATATCCGCAGCAGG

TCTCCAAGGTGAACAGCCTCTGGCATGTTGGAACAATGT

Beat-actin

FP----GAGCACCCTGTGCTGCTCACCGA

RP----CTCTTTGATGTCACGCACGATTTC

345bp

GAGCACCCTGTGCTGCTCACCGAGGCCCCCCTGAACCCTAAGGCCAACCGTGAAAAGATGA

CCCAGATCATGTTTGAGACCTTCAACACCCCAGCCATGTACGTAGCCATCCAGGCTGTGCTG

TCCCTGTATGCCTCTGGTCGTACCACAGGCATTGTGATGGACTCCGGAGACGGGGTCACCCA

CACTGTGCCCATCTACGAGGGCTATGCTCTCCCTCACGCCATCCTGCGTCTGGACCTGGCTGG

CCGGGACCTGACAGACTACCTCATGAAGATCCTGACCGAGCGTGGCTACAGCTTCACCACCA

CAGCTGAGAGGGAAATCGTGCGTGACATCAAAGAG

Peg3 3-6

FP---- ATCCCTGAAACGCTCAAGCCCT

RP---- AAGATCCCGTTGAGGCAGCC

291bp ATCCCTGAAACGCTCAAGCCCTTGGGTGTGAGCAAAACAGACAACTGTGAAAAACTCACCA

CTCCGTTGGAGAGTTTCAAGATGTACCATCACGAAGACGACACCAACAGTGACATGAACAG

TGACGACGACATGAGCCGAAGTGGGAGAGAAACCCCACCCCCTCGACCATCTCATGCTTTTG

GCAGTGAGCGAGACCTGGAGCGCAGGGGCAGAAGCAGAGATGTGGAGCCTCGAGACCGCT

GGCCATACACCAGGAATCCCAGAAGCAGGCTGCCTCAACGGGATCTT
